# Preoperative blood-routine markers and prognosis of esophageal squamous cell carcinoma: The Fujian prospective investigation of cancer (FIESTA) study

**DOI:** 10.18632/oncotarget.13318

**Published:** 2016-11-11

**Authors:** Dan Hu, Xiandong Lin, Yan Chen, Qing Chang, Gang Chen, Chao Li, Hejun Zhang, Zhaolei Cui, Binying Liang, Wenhui Jiang, Kaida Ji, Jun Huang, Feng Peng, Xiongwei Zheng, Wenquan Niu

**Affiliations:** ^1^ Department of Pathology, Fujian Provincial Cancer Hospital, The Affiliated Hospital of Fujian Medical University, Fuzhou, Fujian, China; ^2^ Department of Clinical Laboratory, Fujian Provincial Cancer Hospital, The Affiliated Hospital of Fujian Medical University, Fuzhou, Fujian, China; ^3^ State Key Laboratory of Medical Genomics, Rui Jin Hospital, Shanghai Jiao Tong University School of Medicine, Shanghai, China; ^4^ Department of Medical Record, Fujian Provincial Cancer Hospital, The Affiliated Hospital of Fujian Medical University, Fuzhou, Fujian, China; ^5^ Department of Cardiology, The First Affiliated Hospital of Fujian Medical University, Fuzhou, Fujian, China

**Keywords:** esophageal squamous cell carcinoma, blood-routine marker, prognosis, the FIESTA study

## Abstract

This prospective study was designed to investigate the prognosis of preoperative blood-routine markers for esophageal cancer mortality by using data from the ongoing Fujian prospective investigation of cancer (FIESTA) study. Patients who received three-field lymphadenectomy for esophageal cancer between 2000 and 2010 were enrolled. Of 2535 patients with complete survival data, esophageal squamous cell carcinoma (ESCC) accounted for 94.5% (*n* = 2396). Here, only ESCC patients were analyzed, with the median follow-up time of 38.2 months (range: 0.5 to 180 months). Of 10 blood-routine markers evaluated, platelet count and red cell distribution width (RDW) were two significant predictors for ESCC mortality in men (adjusted hazard ratio or HR = 1.25 and 0.84, 95% confidence interval or CI: 1.08-1.22 and 0.75-0.93, *P* < 0.001 and *P* = 0.001, respectively), while in women only lymphocyte showed marginal significance. Based on individual results, a new derivate calculated as platelet count to RDW ratio (PRR) was created, and it was superior over other widely-evaluated derivates in men after adjustment (HR = 1.21, 95% CI: 1.13-1.30, *P* < 0.001), while there was no observable significance in women. In further stratified analyses, the prognosis of PRR for ESCC mortality was reinforced in men with tumor-node-metastasis stage III (HR, 95% CI, P: 1.18, 1.09-1.28, 0.001), invasion depth T3-T4 (1.17, 1.08-1.26, <0.001) or positive lymph node metastasis (1.37, 1.18-1.59, <0.001). Taken together, we created a new derivate PRR that was proven to be superior over other blood-routine markers and exhibited strong prognostic capability for ESCC mortality in Chinese men.

## INTRODUCTION

Esophageal cancer ranks as the 4^th^ most frequent cancer in China, as projected by the latest national statistics reporting a large proportion in cancer-related incidence (11.1%) and mortality (13.3%) in 2015 [1]. Due to the gender-specific pathogenic factors, men are 3 to 4 times more likely to experience esophageal cancer than women [2]. Esophageal cancer is a highly aggressive digestive malignancy, and is featured by rapid growth and early metastasis. It is estimated that distant metastasis rate was 20-30% in esophageal cancer patients at the time of initial diagnosis [3]. Esophageal squamous cell carcinoma (ESCC) and esophageal adenocarcinoma (EAC) constitute two major histological types of esophageal cancer. More concerning is their epidemiologic disproportionation, as compared with the most prevalent EAC in many Western countries, ESCC is predominant in Chinese, accounting for over 90% of esophageal cancer cases [4, 5]. Despite the dramatic advancements made in cancer management, the 5-year overall survival rate of esophageal cancer is around 20% in China, highlighting a urgent need to seek effective biomarkers with prognostic significance to guide treatment [6, 7].

Use of noninvasive easy-to-obtain markers is proposed as a more practical approach toward clinical translational applications. There is increasing recognition that some blood-routine markers are strong prognostic indicators in patients with esophageal cancer [8–11]. For instance, platelet count and mean platelet volume were identified as promising predictors for the postoperative survival in ESCC patients [9]. As evidenced by a recent meta-analysis, the neutrophil-to-lymphocyte ratio and platelet-to-lymphocyte ratio can predict worse survival in patients with esophageal cancer [12]. However, these findings have not been confirmed uniformly, and the impact of blood-routine markers on esophageal carcinogenesis is still subject to an ongoing debate and no consensus has been reached yet. It is worth noting that the majority of studies in this field are retrospective in design and individually underpowered [13–15], and thus they may suffer from confounding bias or chance false-positive error. Consequently, we used a subset of data on esophageal cancer from the Fujian prospective investigation of cancer (FIESTA) study, and over a 15-year follow-up we sought to investigate the prognosis of preoperative blood-routine markers for esophageal cancer mortality.

The FIESTA study started in January 2000, and it is an ongoing prospective cohort study of common digestive system tumors in China, including esophageal cancer, gastric cancer and colorectal cancer [16, 17]. The FIESTA study was designed to look for preoperative prognostic factors for cancer-specific mortality, aiming to help delay tumor progression and prolong the survival of cancer patients after the surgery.

## MATERIALS AND METHODS

### Study patients

A total of 2886 patients who consecutively received three-field lymphadenectomy for esophageal cancer at the Department of Thoracic Surgery, Fujian Provincial Cancer Hospital between January 2000 and December 2010 and survived the hospitalization with complete follow-up data were enrolled. The conduct of the FIESTA study was approved by the Ethics Committee of Fujian Provincial Cancer Hospital. All enrolled patients completed written informed consent.

### Eligibility criteria

The diagnosis of esophageal cancer is confirmed by preoperative biopsy or postoperative pathologic examination. Patients were included if they were of Han nationality and firstly hospitalized for esophageal cancer surgery, as well as they had no history of malignancies and received no preoperative and postoperative chemotherapy or radiotherapy.

### Tissue collection

A pair of esophageal cancer and nearby normal tissues were cut from each patient, and then fixed with 10% neutral formalin embedded in paraffin. Clinicopathological analyses of tissue samples were completed at the Department of Pathology, Fujian Provincial Cancer Hospital.

### Follow-up assessment

Clinical outcomes were assessed every 6 to 12 months after discharge at the Out-Patient Department or via calling or posting mails in case of no-show at the reserved time. All patients were followed up from the initiation of this study in January 2000 until death or the last checkpoint during the year 2015, whichever came first. Over a 15-year period, 1265 deaths from esophageal cancer occurred, leaving 1207 survivors.

### Blood-routine markers

Qualified patients who had normal diets were requested to be fasted after midnight prior to the surgery. On the morning of surgery day, fasting (at least 8 hours) venous blood sample (4 mL) was drawn into the EDTA-K2 anticoagulative tubes to measure neutrophil, lymphocyte, monocyte, eosinophil, basophil, white blood cell, red blood cell, hemoglobin, red cell distribution width (RDW) and platelet count by the SYSMEX XE-2100 (Sysmex, Kobe, Japan) Automatic Blood Cell Analyzer. The whole process from sample collection to clinical measurement was conducted according to standard procedures and was completed within 4 hours. Classic blood-routine derivates including neutrophil-to-lymphocyte ratio (NLR), platelet-to-lymphocyte ratio (PLR) and lymphocyte-to-monocyte ratio (LMR) were also calculated. In addition, a new derivate, platelet count to RDW ratio namely PRR, was created based on the results of single blood-routine markers.

### Baseline characteristics

At enrollment, each qualified patient was requested to complete a self-designed questionnaire gathering information on socio-demographic and anthropometric characteristics, including date of birth, age of onset, gender, body weight, height, smoking, drinking and family cancer history. Age was defined as the age at the time of operation for esophageal cancer. Body weight and height were measured when patients were in light clothing and with bare feet, and body mass index was accordingly calculated as body weight divided by height in meters-squared (kg/m^2^). Smoking status was classified as never smoking and ever smoking (including former and current smoking). Drinking status was classified as never drinking and ever drinking (including former and current drinking). A positive family cancer history referred to one or more of affected relatives within three generations who suffered malignancies except for non-melanoma skin tumor.

Blood pressure was measured with a conventional mercury sphygmomanometer on three occasions of at least 5 min intervals by certified examiners according to a standard protocol recommended by the American Heart Association [18]. Fasting blood glucose was measured by an automated glucose oxidase method.

From pathological reports, clinicopathologic features were extracted, including histological type (ESCC, EAC and esophageal neuroendocrine carcinomas), tumor size (in centimeters), tumor-node-metastasis (TNM) stage (I, II, III and IV according to the 7^th^ Edition of the UICC/AJCC TNM Staging System [19]), invasion depth (T1, T2, T3 and T4), regional lymph node metastasis or LNM (N0, N1, N2 and N3), distant metastasis (M0 and M1), tumor location (upper, middle and lower esophagus), histological differentiation (well, moderate and poor differentiation), tumor embolus (positivity) and tumor size.

### Statistical analysis

Continuous data are expressed as mean ± standard deviation (s.d.) and categorical data as percentage. Differences between genders were determined by unpaired t test or the χ^2^ test where appropriate. All blood-routine markers and derivates were analyzed as continuous variables and all clinicopathologic features were analyzed as categorical variables. Kaplan-Meier curves and Log-rank tests were used to detect survival differences across different characteristics. Receiver operating characteristic (ROC) analysis was used to calculate area under the curve. Adjusted risk estimates (hazard ratio or HR and 95% confidence interval or CI) were calculated using multivariate Weibull proportional hazards regression analyses. All statistical tests were two-sided, and a probability < 0.05 was considered to be statistically significant. All analyses were managed with STATA for Windows (StataCorp, TX, USA, version 13.0).

## RESULTS

### Follow-up observation

Over a 15-year period, 147 patients were lost to follow-up and 204 patients died of causes other than esophageal cancer as of December 31, 2015, leaving 2535 assessable patients aged 30 to 88 years (1929 men and 606 women). The 5-year survival rate was 52.2%, which was comparable with that of previous reports [20, 21]. According to the histological types, there are 2396 patients with ESCC, 83 patients with EAC and 56 patients with esophageal neuroendocrine carcinomas. The Kaplan-Meier curves of three histological types are depicted in Supplementary Figure S1. The median survival time was 111.1 months for ESCC patients, 52.2 months for EAC patients and 32.4 months for patients with esophageal neuroendocrine carcinomas, and the difference was statistically significant (Log-rank test: *P* = 0.0082). Due to the small number of patients with EAC and esophageal neuroendocrine carcinomas, our analysis was restricted to ESCC patients only, including 1822 men and 574 women. The median follow-up time of 2396 ESCC patients was 38.2 months (range: 0.5 to 180 months).

### Baseline characteristics

Table 1 shows the baseline characteristics of cohort ESCC patients by gender. Male patients tended to be younger and leaner, and had a higher proportion of ever smokers and ever drinkers (*P* < 0.001). The proportion of positive family cancer history was slightly higher in men than in women (*P* = 0.026). Systolic and diastolic blood pressure and fasting glucose did not differ significantly between genders. The median levels of neutrophil, monocyte, white blood cell, hemoglobin, NLR, LMR and tumor size were significantly higher in men than in women (*P* < 0.001), while that of PRR were slightly lower (*P* = 0.023). In addition, the distributions of invasion depth, regional lymph node metastasis and TNM stage differed significantly between genders (*P* < 0.001).

**Table 1:** The baseline characteristics of cohort patients with esophageal squamous cell carcinoma by gender

Characteristics	Men (*n*= 1822)	Women (*n*= 574)	*P*
Age (years)	55.98±9.81	57.93±9.41	<0.001
Body mass index (kg/m^2^)	22.14±2.90	22.83±3.26	<0.001
Ever smoking	986 (54.12%)	16 (2.79%)	<0.001
Ever drinking	473 (25.96%)	8 (1.39%)	<0.001
Positive family cancer history	268 (14.71%)	63 (10.98%)	0.026
SBP (mmHg)	123.74±18.22	125.33±18.28	0.070
DBP (mmHg)	77.34±10.43	78.24±10.73	0.073
Fasting blood glucose (mmol/L)	6.04±2.52	6.22±2.53	0.142
Neutrophil (10^9^/L)	4.00 (3.10, 5.10)	3.30 (2.55, 4.30)	<0.001
Lymphocyte (10^9^/L)	2.00 (1.50, 2.40)	2.00 (1.60, 2.40)	0.960
Monocyte (10^9^/L)	0.60 (0.50, 0.70)	0.50 (0.40, 0.60)	<0.001
Eosinophil (10^9^/L)	0.20 (0.10, 0.30)	0.10 (0.10, 0.30)	0.492
Basophil (10^9^/L)	0 (0, 0.1)	0 (0, 0)	0.322
White blood cell (10^9^/L)	6.90 (5.80, 8.30)	6.20 (5.10, 7.50)	<0.001
Red blood cell (10^12^/L)	4.40 (4.10, 4.70)	4.20 (3.91, 4.44)	0.470
Hemoglobin (g/L)	139 (129, 148)	128 (119, 134)	<0.001
RDW (%)	12.90 (12.30, 13.70)	12.70 (12.20, 13.40)	0.261
Platelet (10^9^/L)	238 (198, 287)	246 (204, 285)	0.246
NLR	2.09 (1.52, 2.91)	1.64 (1.22, 2.38)	<0.001
PLR	123.48 (92.86, 160.00)	124.29 (94.84, 160.91)	0.258
LMR	3.25 (2.50, 4.17)	4.25 (3.20, 5.50)	<0.001
PRR	18.48 (15.06, 22.21)	19.19 (15.91, 22.29)	0.023
Esophagus location			0.205
Upper	179 (9.82%)	60 (10.45%)	
Middle	1453 (79.75%)	469 (81.71%)	
Lower	190 (10.43%)	44 (7.67%)	
Histological differentiation			0.838
Well	278 (15.26%)	84 (14.63%)	
Moderate	1216 (66.74%)	381 (66.38%)	
Poor	328 (18.00%)	109 (18.99%)	
Invasion depth			<0.001
T1-T2	447 (24.53%)	233 (40.59%)	
T3-T4	1375 (75.47%)	341 (59.41%)	
Regional lymph node metastasis			<0.001
N0	710 (38.97%)	286 (49.83%)	
N1	522 (28.65%)	161 (28.05%)	
N2	383 (21.02%)	92 (16.03%)	
N3	207 (11.36%)	35 (6.10%)	
Tumor embolus (+)	312 (17.12%)	81 (14.11%)	0.089
TNM stage			<0.001
I	140 (7.68%)	83 (14.46%)	
II	560 (30.74%)	221 (38.50%)	
III	1123 (61.64%)	270 (47.04%)	
Tumor size (cm)	4.50 (3.00, 6.00)	4.00 (2.80, 5.00)	<0.001

Notes: SBP, systolic blood pressure; DBP, diastolic blood pressure; RDW, red cell distribution width; NLR, neutrophil-to-lymphocyte ratio; PLR, platelet-to-lymphocyte ratio; LMR, lymphocyte-to-monocyte ratio; PRR, platelet count to RDW ratio. Data are expressed as mean±standard deviation or median (interquartile range) or number(percentage). P was calculated by the t-test or Mann-Whitney U Test or Chisq test where appropriate.

### Overall prediction of blood-routine markers

In view of gender-specific differences, the prediction of preoperative blood-routine markers for ESCC mortality was classified by genders, with and without adjusting for age, body mass index, smoking, drinking, family history of cancer, systolic blood pressure, fasting blood glucose, TNM stage, tumor embolus and tumor size (Table 2). On the one hand, for single blood-routine markers, in men per s.d. increments in neutrophil and platelet count were significantly associated with poor survival of ESCC patients, while per s.d. increments in lymphocyte and RDW improved overall survival, even after adjustment, and significance was strikingly significant for RDW and platelet count (adjusted HR = 0.84 and 1.25, 95% CI: 0.75-0.93 and 1.08-1.22, *P* = 0.001 and < 0.001, respectively). In women, only marginal significance was noted for lymphocyte with improved survival after adjustment. Based on above findings, a new prognostic derivate calculated as platelet count to RDW ratio (PRR) was therefore created.

**Table 2:** Single blood routine markers and derives in overall association with the risk of esophageal squamous cell carcinoma mortality

Blood parameters	Men		Women	
	HR, 95% CI, *P*	HR, 95% CI, P*	HR, 95% CI, *P*	HR, 95% CI, P*
***Single blood-routine markers***				
Neutrophil	1.10, 1.03-1.17, 0.005	1.09, 1.02-1.16, 0.014	1.04, 0.90-1.20, 0.572	1.05, 0.92-1.21, 0.465
Lymphocyte	0.91, 0.85-0.99, 0.019	0.92, 0.85-0.99, 0.032	0.89, 0.76-1.05, 0.162	0.84, 0.72-0.99, 0.037
Monocyte	0.93, 0.86-1.01, 0.098	0.92, 0.85-1.00, 0.053	1.00, 0.84-1.19, 0.968	1.01, 0.86-1.19, 0.882
Eosinophil	0.92, 0.84-1.01, 0.072	0.91, 0.83-0.99, 0.039	0.94, 0.80-1.17, 0.493	0.93, 0.78-1.10, 0.399
Basophil	0.93, 0.84-1.04, 0.210	0.94, 0.85-1.05, 0.292	1.01, 0.88-1.15, 0.939	1.01, 0.87-1.16, 0.936
White blood cell count	0.99, 0.92-1.06, 0.794	0.98, 0.91-1.06, 0.676	1.01, 0.86-1.16, 0.994	1.01, 0.87-1.17, 0.838
Red blood cell count	0.99, 0.92-1.06, 0.723	1.00, 0.93-1.08, 0.964	1.00, 0.86-1.16, 0.994	1.00, 0.87-1.16, 0.963
Hemoglobin	1.07, 0.99-1.15, 0.093	1.08, 1.00-1.17, 0.048	0.97, 0.83-1.12, 0.662	1.00, 0.86-1.17, 0.996
RDW	0.85, 0.76-0.94, 0.002	0.84, 0.75-0.93, 0.001	1.02, 0.89-1.18, 0.730	1.01, 0.88-1.17, 0.996
Platelet count	1.23, 1.14-1.32, <0.001	1.25, 1.16-1.35, <0.001	0.85, 0.74-0.99, 0.040	0.90, 0.78-1.05, 0.192
***Blood-routine derivates***				
NLR	1.06, 1.00-1.17, 0.045	1.05, 0.99-1.11, 0.119	1.06, 0.94-1.20, 0.312	1.08, 0.96-1.20, 0.199
PLR	1.16, 1.09-1.23, <0.001	1.15, 1.08-1.22, <0.001	0.91, 0.76-1.10, 0.346	0.98, 0.84-1.15, 0.844
LMR	0.94, 0.86-1.02, 0.127	0.95, 0.87-1.03, 0.201	0.85, 0.66-1.10, 0.224	0.78, 0.58-1.05, 0.104
PRR	1.18, 1.10-1.27, <0.001	1.21, 1.13-1.30, <0.001	0.86, 0.73-1.01, 0.074	0.92, 0.78-1.09, 0.329

Notes: RDW, red cell distribution width; NLR, neutrophil-to-lymphocyte ratio; PLR, platelet-to-lymphocyte ratio; LMR, lymphocyte-to-monocyte ratio; PRR, platelet count to RDW ratio; HR, hazard ratio; 95% CI, 95% confidence interval. *P was adjusted for age, body mass index, smoking, drinking, family history of cancer, systolic blood pressure, fasting blood glucose, TNM stage, tumor embolus and tumor size.

On the other hand, the prognosis of PRR for ESCC mortality was compared with three classic blood-routine derivates (NLR, PLR and LMR), and it is interesting to find that PRR was superior over these three derivates in men after adjustment (per s.d. increment: HR = 1.21, 95% CI: 1.13-1.30, *P* < 0.001), while there was no observable significance in women. Further ROC analysis indicated that in men area under the curve was the largest for PRR (0.571, 95% CI: 0.543-0.600), relative to NLR (0.553, 95% CI: 0.525-0.582), PLR (0.555, 95% CI: 0.526-0.584) and LMR (0.482, 95% CI: 0.453-0.510).

### Stratified prediction of PRR

The prognosis of preoperative PRR for ESCC mortality was stratified by clinicopathologic characteristics, including TNM stage, invasion depth, regional LNM, tumor embolus and tumor size (Table 3). In men, the HRs were statistically significant in ESCC patients with TNM stage III (HR, 95% CI, P: 1.18, 1.09-1.28, 0.001), invasion depth T3-T4 (HR, 95% CI, P: 1.17, 1.08-1.26, < 0.001) and regional LNM N1-N3 (HR, 95% CI, P: 1.18, 1.09-1.28, < 0.001), as well as with both positive (HR, 95% CI, P: 1.37, 1.18-1.59, < 0.001) and negative (HR, 95% CI, P: 1.19, 1.09-1.29, < 0.001) tumor embolus, small (HR, 95% CI, P: 1.24, 1.10-1.39, < 0.001) and large (HR, 95% CI, P: 1.18, 1.07-1.30, 0.001) tumors after adjusting for age, body mass index, smoking, drinking, family history of cancer, systolic blood pressure, fasting blood glucose. In women, PRR was associated with a reduced risk of ESCC mortality and significance was only noticed in patients with positive tumor embolus.

**Table 3:** PRR in stratified association with the risk of esophageal squamous cell carcinoma mortality

PRR	Men		Women	
	HR, 95% CI, *P*	HR, 95% CI, P*	HR, 95% CI, *P*	HR, 95% CI, P*
TNM stage (I-II)	1.03, 0.88-1.21, 0.700	1.08, 0.92-1.27, 0.352	0.79, 0.56-1.13, 0.192	0.88, 0.62-1.25, 0.467
TNM stage (III)	1.15, 1.07-1.25, 0.002	1.18, 1.09-1.28, 0.001	0.89, 0.74-1.07, 0.226	0.93, 0.77-1.11, 0.411
Invasion depth (T1-T2)	1.18, 0.96-1.44, 0.114	1.20, 0.98-1.47, 0.077	0.83, 0.59-1.18, 0.304	0.88, 0.63-1.24, 0.469
Invasion depth (T3-T4)	1.14, 1.06-1.23, 0.001	1.17, 1.08-1.26, <0.001	0.87, 0.72-1.05, 0.155	0.94, 0.78-1.14, 0.536
LNM (N0)	1.09, 0.94-1.27, 0.248	1.14, 0.98-1.34, 0.090	0.96, 0.66-1.38, 0.808	1.03, 0.72-1.47, 0.864
LNM (N1-N3)	1.16, 1.07-1.25, <0.001	1.18, 1.09-1.28, <0.001	0.88, 0.74-1.05, 0.162	0.91, 0.77-1.09, 0.301
Tumor embolus (−)	1.16, 1.07-1.26, <0.001	1.19, 1.09-1.29, <0.001	0.90, 0.74-1.09, 0.277	0.97, 0.80-1.17, 0.724
Tumor embolus (+)	1.37, 1.19-1.58, <0.001	1.37, 1.18-1.59, <0.001	0.71, 0.51-0.99, 0.044	0.74, 0.53-1.03, 0.078
Tumor size ≤ 4.5 cm	1.16, 1.04-1.30, 0.007	1.24, 1.10-1.39, <0.001	0.82, 0.65-1.03, 0.095	0.91, 0.72-1.17, 0.473
Tumor size > 4.5 cm	1.16, 1.06-1.28, 0.002	1.18, 1.07-1.30, 0.001	0.87, 0.70-1.09, 0.236	0.89, 0.72-1.11, 0.294

Notes: PRR, platelet count to RDW ratio; TNM, tumor-node-metastasis; HR, hazard ratio; 95% CI, 95% confidence interval; LNM, regional lymph node metastasis. *P was adjusted for age, body mass index, smoking, drinking, family history of cancer, systolic blood pressure and fasting blood glucose.

## DISCUSSION

The most noteworthy finding of this prospective study was that we created a new derivate PRR that was superior over other blood-routine markers and exhibited strong prognostic capability for ESCC mortality in Chinese men. Moreover, the prognosis of preoperative PRR was strongly potentiated in male patients with TNM stage III, invasion depth III-IV and positive lymph node metastasis. To the authors’ knowledge, this is so far the largest prospective study of 2396 ESCC patients with a median follow-up of 38.2 months that has investigated the prognosis of preoperative blood-routine markers for esophageal cancer mortality in medical literature.

A growing number of epidemiological studies have underscored the clinical importance of platelet count and RDW in the diagnosis or prognosis of many malignancies [9, 22–26]. Platelet can be activated by inflammatory factors such as interleukin 6, and elevated platelet count in peripheral blood is associated to the onset and progress of many cancers [9, 27–29]. There is compelling evidence that platelet activation is identified as a crucial biological process in carcinogenesis and metastasis [30]. Elevated platelet activation leads to hyper-coagulation activity, which can precipitate the formation of embolus in tumor endothelial cells and secrete vascular endothelial growth factors, speeding up tumor angiogenesis and metastasis [31]. As expected in the present study, we in a large prospective study confirmed the prognostic utility of platelet count in ESCC patients, consistent with the findings of other studies [9, 22]. Likewise, RDW measures the variability of red blood cell size and volume, and several studies suggested that RDW was associated with oxidative stress and inflammation [32, 33], the two conditions being closely linked to carcinogenesis [34]. Inflammation in turn may impair red blood cell maturation by damaging cell membrane, causing increased RDW [35]. However, in this study, we surprisingly found that elevated RDW was associated with significant better prognosis of ESCC in men, opposing to the findings of most existing studies in cancer field [36–40]. Before going into this seeming contradiction, it is worth mentioning the conclusion of the Malmo Diet and Cancer cohort involving 26709 non-diabetic adults over 14 years of follow-up, that is, low RDW was significantly associated with increased incidence of diabetes mellitus, and this association was independent of traditional risk factors [41]. In fact, aerobic glycolysis is proposed as a cancer hallmark and elevated glucose consumption is a necessary component of carcinogenesis [42]. It is therefore reasonable to speculate that elevated RDW may be a surrogate indicator of improved glucose metabolism, which is the key for good survival of ESCC. Although elucidating the underlying mechanisms between elevated RDW and improved ESCC prognosis is beyond the scope of the present study, this speculation should be confirmed physiologically or epidemiologically in larger cohorts with longer follow-up.

In view of the opposite prediction between platelet count and RDW for the prognosis of ESCC, we therefore created a new derivate PRR. A note of caution should be sounded in view of individual results of RDW and platelet count, the stronger prognostic capability of PRR seen in men is within our expectation, while its role in older women seems counterintuitive. There are several possible explanations. First, it might be due to divergent risk profiles associated with both genders [2]. For example, abdominal is more common in men [43], leading us to speculate that such adiposity explains some gender-specific divergences in cancer risk. Second, sex hormones might also account for the discrimination ability of PRR for ESCC mortality, especially for women before and after menopause, as serum estrogen concentrations are significantly reduced in postmenopausal women [44, 45]. The third explanation lies in unbalanced statistical power as the number of men has almost quadrupled that of women in our prospective cohort. We agree that validation of this gender-specific distinction for PRR in a larger cohort of women is critical. Nevertheless, our findings highlight the gender-specific prognostic importance of PRR in identifying high-risk ESCC patients.

Another key finding of this study is the prognosis of PRR for ESCC mortality was more prominent in men with TNM stage III, invasion depth III-IV or positive lymph node metastasis in our stratified analyses. Esophageal cancer is a highly aggressive digestive malignancy, and most patients are diagnosed at an advanced stage, and about 30% of them have distant metastasis [3]. Importantly, the prognostic utility of PRR for ESCC mortality is superior to that of NLR, PLR and LMR in this study. Although to unravel the precise mechanisms underlying this finding transcends the limits of this study, we postulate that altered PRR might be a strong indicator of chronic inflammation or hyperglycemia, which participates in providing conditions that trigger carcinogenesis and metastasis.

Several limitations should be acknowledged. First, other blood-routine markers such as mean platelet volume are unavailable in this study. It is widely reorganized that mean platelet volume is superior over platelet count as a marker of platelet function and activation [46, 47]. Second, the findings presented in this study cannot be directly extrapolated to the general populations as we only enrolled postoperative patients for esophageal cancer. Third, although this is so far the largest prospective study for ESCC mortality, the number of patients diagnosed as EAC or esophageal neuroendocrine carcinomas is small, limiting further exploratory data analyses. Future studies with large sample sizes specifically designed to examine the prognosis of blood-routine markers or derivates in patients with these two rare types are warranted.

Taken together, we created a new derivate PRR that was superior over other blood-routine markers and exhibited strong prognostic capability for ESCC mortality with a median follow-up of 38.2 months, especially in Chinese men. Moreover, elevated PRR was associated with worse prognosis in male patients especially at an advanced stage and with distant metastasis. Knowledge revolving the etiology of platelet count and RDW in ESCC survivors will be of clinical importance to improve their prognosis after surgery and further formulate more targeted and effective management strategies for high risk patients.

## SUPPLEMENTARY MATERIAL FIGURE



## References

[1] Chen W, Zheng R, Baade PD, Zhang S, Zeng H, Bray F, Jemal A, Yu XQ, He J (2016). Cancer statistics in China, 2015. CA Cancer J Clin.

[2] Rustgi AK, El-Serag HB (2014). Esophageal carcinoma. N Engl J Med.

[3] Napier KJ, Scheerer M, Misra S (2014). Esophageal cancer: A Review of epidemiology, pathogenesis, staging workup and treatment modalities. World J Gastrointest Oncol.

[4] Torre LA, Bray F, Siegel RL, Ferlay J, Lortet-Tieulent J, Jemal A (2015). Global cancer statistics, 2012. CA Cancer J Clin.

[5] Arnold M, Soerjomataram I, Ferlay J, Forman D (2015). Global incidence of oesophageal cancer by histological subtype in 2012. Gut.

[6] Zeng H, Zheng R, Guo Y, Zhang S, Zou X, Wang N, Zhang L, Tang J, Chen J, Wei K, Huang S, Wang J, Yu L (2015). Cancer survival in China, 2003-2005: a population-based study. Int J Cancer.

[7] Tseng RC, Chang JM, Chen JH, Huang WR, Tang YA, Kuo IY, Yan JJ, Lai WW, Wang YC (2015). Deregulation of SLIT2-mediated Cdc42 activity is associated with esophageal cancer metastasis and poor prognosis. J Thorac Oncol.

[8] Kosumi K, Baba Y, Ishimoto T, Harada K, Nakamura K, Ohuchi M, Kiyozumi Y, Izumi D, Tokunaga R, Taki K, Higashi T, Miyata T, Kurashige J (2016). Neutrophil/lymphocyte ratio predicts the prognosis in esophageal squamous cell carcinoma patients. Surg Today.

[9] Zhang F, Chen Z, Wang P, Hu X, Gao Y, He J (2016). Combination of platelet count and mean platelet volume (COP-MPV) predicts postoperative prognosis in both resectable early and advanced stage esophageal squamous cell cancer patients. Tumour Biol.

[10] Zhang J, Zhu Z, Liu Y, Jin X, Xu Z, Yu Q, Li K (2015). Diagnostic value of multiple tumor markers for patients with esophageal carcinoma. PLoS One.

[11] Wang S, Xiao T, Hu C (2016). Are preoperative platelet-lymphocyte and neutrophil-lymphocyte ratio prognostic factors for patients with esophageal squamous cell cancer?. Dis Esophagus.

[12] Yodying H, Matsuda A, Miyashita M, Matsumoto S, Sakurazawa N, Yamada M, Uchida E (2016). Prognostic Significance of Neutrophil-to-Lymphocyte Ratio and Platelet-to-Lymphocyte Ratio in Oncologic Outcomes of Esophageal Cancer: A Systematic Review and Meta-analysis. Ann Surg Oncol.

[13] Zhang F, Chen Z, Wang P, Hu X, Gao Y, He J (2016). Combination of platelet count and mean platelet volume (COP-MPV) predicts postoperative prognosis in both resectable early and advanced stage esophageal squamous cell cancer patients. Tumour Biol.

[14] Yutong H, Xiaoli X, Shumei L, Shan S, Di L, Baoen S (2015). Increased Neutrophil-Lymphocyte Ratio Is a Poor Prognostic Factor in Patients with Esophageal Cancer in a High Incidence Area in China. Arch Med Res.

[15] Xie X, Luo KJ, Hu Y, Wang JY, Chen J (2016). Prognostic value of preoperative platelet-lymphocyte and neutrophil-lymphocyte ratio in patients undergoing surgery for esophageal squamous cell cancer. Dis Esophagus.

[16] Hu D, Peng F, Lin X, Chen G, Liang B, Li C, Zhang H, Liao X, Lin J, Zheng X, Niu W (2016). The elevated preoperative fasting blood glucose predicts a poor prognosis in patients with esophageal squamous cell carcinoma: The fujian prospective investigation of cancer (FIESTA) study. Oncotarget.

[17] Peng F, Hu D, Lin X, Chen G, Liang B, Zhang H, Ji K, Huang J, Lin J, Zheng X, Niu W (2016). Preoperative metabolic syndrome and prognosis after radical resection for colorectal cancer: The Fujian prospective investigation of cancer (FIESTA) study. Int J Cancer.

[18] Perloff D, Grim C, Flack J, Frohlich ED, Hill M, McDonald M, Morgenstern BZ (1993). Human blood pressure determination by sphygmomanometry. Circulation.

[19] Edge SB, Compton CC (2010). The American Joint Committee on Cancer: the 7th edition of the AJCC cancer staging manual and the future of TNM. Ann Surg Oncol.

[20] Kang CH, Kim YT, Jeon SH, Sung SW, Kim JH (2007). Lymphadenectomy extent is closely related to long-term survival in esophageal cancer. Eur J Cardiothorac Surg.

[21] Nishimaki T, Suzuki T, Kanda T, Obinata I, Komukai S, Hatakeyama K (1999). Extended radical esophagectomy for superficially invasive carcinoma of the esophagus. Surgery.

[22] Feng JF, Huang Y, Chen QX (2014). The combination of platelet count and neutrophil lymphocyte ratio is a predictive factor in patients with esophageal squamous cell carcinoma. Transl Oncol.

[23] Kao CH, Lin SC, Hsieh TC, Yen KY, Yang SN, Wang YC, Liang JA, Hua CH, Chen SW (2012). Use of pretreatment metabolic tumour volumes to predict the outcome of pharyngeal cancer treated by definitive radiotherapy. Eur J Nucl Med Mol Imaging.

[24] Lian L, Xia YY, Zhou C, Shen XM, Li XL, Han SG, Zheng Y, Mao ZQ, Gong FR, Wu MY, Chen K, Tao M, Li W (2015). Application of platelet/lymphocyte and neutrophil/lymphocyte ratios in early diagnosis and prognostic prediction in patients with resectable gastric cancer. Cancer Biomark.

[25] Oermann EK, Wu J, Guan KL, Xiong Y (2012). Alterations of metabolic genes and metabolites in cancer. Semin Cell Dev Biol.

[26] Cao MD, Giskeodegard GF, Bathen TF, Sitter B, Bofin A, Lonning PE, Lundgren S, Gribbestad IS (2012). Prognostic value of metabolic response in breast cancer patients receiving neoadjuvant chemotherapy. BMC Cancer.

[27] Berstein LM, Tsyrlina EV, Semiglazov VF, Kovalenko IG, Gamayunova VB, Evtushenko TP, Ivanova OA (1997). Hormone-metabolic status in moderately smoking breast cancer patients. Acta Oncol.

[28] Chlebowski RT, Heber D (1986). Metabolic abnormalities in cancer patients: carbohydrate metabolism. Surg Clin North Am.

[29] Huang S, Chong N, Lewis NE, Jia W, Xie G, Garmire LX (2016). Novel personalized pathway-based metabolomics models reveal key metabolic pathways for breast cancer diagnosis. Genome Med.

[30] Chen BB, Tien YW, Chang MC, Cheng MF, Chang YT, Wu CH, Chen XJ, Kuo TC, Yang SH, Shih IL, Lai HS, Shih TT (2016). PET/MRI in pancreatic and periampullary cancer: correlating diffusion-weighted imaging, MR spectroscopy and glucose metabolic activity with clinical stage and prognosis. Eur J Nucl Med Mol Imaging.

[31] Ikeda M, Furukawa H, Imamura H, Shimizu J, Ishida H, Masutani S, Tatsuta M, Satomi T (2002). Poor prognosis associated with thrombocytosis in patients with gastric cancer. Ann Surg Oncol.

[32] Jhaveri K, Ulaner GA, Dickler MN (2015). Predictive Value of Positron Emission Tomography/Computed Tomography to Assess Early Treatment Response to Dual Human Epidermal Growth Factor Receptor 2 (HER2) Blockade Without Chemotherapy for HER2-Positive Metastatic Breast Cancer: Are We Ready to Embrace This “Early Metabolic Look” Strategy?. J Clin Oncol.

[33] Son SH, Lee SW, Jeong SY, Song BI, Chae YS, Ahn BC, Lee J (2015). Whole-Body Metabolic Tumor Volume, as Determined by (18)F-FDG PET/CT, as a Prognostic Factor of Outcome for Patients With Breast Cancer Who Have Distant Metastasis. AJR Am J Roentgenol.

[34] Lohsiriwat V, Pongsanguansuk W, Lertakyamanee N, Lohsiriwat D (2010). Impact of metabolic syndrome on the short-term outcomes of colorectal cancer surgery. Dis Colon Rectum.

[35] Krawczyk J, Kraj L, Ziarkiewicz M, Wiktor-Jedrzejczak W (2014). Metabolic and nutritional aspects of cancer. Postepy Hig Med Dosw (Online).

[36] Ellingsen TS, Lappegard J, Skjelbakken T, Braekkan SK, Hansen JB (2015). Impact of red cell distribution width on future risk of cancer and all-cause mortality among cancer patients - the Tromso Study. Haematologica.

[37] Albayrak S, Zengin K, Tanik S, Bakirtas H, Imamoglu A, Gurdal M (2014). Red cell distribution width as a predictor of prostate cancer progression. Asian Pac J Cancer Prev.

[38] Balta S, Arslan Z, Unlu M, Demirkol S (2014). The association between red cell distribution width and non-small-cell lung cancer. Eur J Cardiothorac Surg.

[39] Seretis C, Seretis F, Lagoudianakis E, Gemenetzis G, Salemis NS (2013). Is red cell distribution width a novel biomarker of breast cancer activity? Data from a pilot study. J Clin Med Res.

[40] Warwick R, Mediratta N, Shackcloth M, Shaw M, McShane J, Poullis M (2014). Preoperative red cell distribution width in patients undergoing pulmonary resections for non-small-cell lung cancer. Eur J Cardiothorac Surg.

[41] Engstrom G, Smith JG, Persson M, Nilsson PM, Melander O, Hedblad B (2014). Red cell distribution width, haemoglobin A1c and incidence of diabetes mellitus. J Intern Med.

[42] Gillies RJ, Robey I, Gatenby RA (2008). Causes and consequences of increased glucose metabolism of cancers. J Nucl Med.

[43] Isidoro A, Casado E, Redondo A, Acebo P, Espinosa E, Alonso AM, Cejas P, Hardisson D, Fresno Vara JA, Belda-Iniesta C, Gonzalez-Baron M, Cuezva JM (2005). Breast carcinomas fulfill the Warburg hypothesis and provide metabolic markers of cancer prognosis. Carcinogenesis.

[44] Beloribi-Djefaflia S, Vasseur S, Guillaumond F (2016). Lipid metabolic reprogramming in cancer cells. Oncogenesis.

[45] Lai CH (2016). Measuring tumor metabolic heterogeneity on positron emission tomography: utility in cervical cancer. J Gynecol Oncol.

[46] Yilmaz A, Mohamed N, Patterson KA, Tang Y, Shilo K, Villalona-Calero MA, Davis ME, Zhou XP, Frankel W, Otterson GA, Zhao W (2014). Clinical and metabolic parameters in non-small cell lung carcinoma and colorectal cancer patients with and without KRAS mutations. Int J Environ Res Public Health.

[47] Santoro M, Guido C, De Amicis F, Sisci D, Cione E, Vincenza D, Dona A, Panno ML, Aquila S (2016). Bergapten induces metabolic reprogramming in breast cancer cells. Oncol Rep.

