# Supplementary material for: Preoperative blood-routine markers and prognosis of esophageal squamous cell carcinoma: The Fujian prospective investigation of cancer (FIESTA) study
Source: Oncotarget. 2016 Nov 11;8(14):23841–50. doi: 10.18632/oncotarget.13318 (PMC5410348; doi:10.18632/oncotarget.13318)
Supplement: Supplementary file 1 [file oncotarget-08-23841-s001.pdf]

Preoperative blood-routine markers and prognosis of esophageal squamous cell carcinoma: The Fujian prospective investigation of cancer (FIESTA) study

Supplementary Material

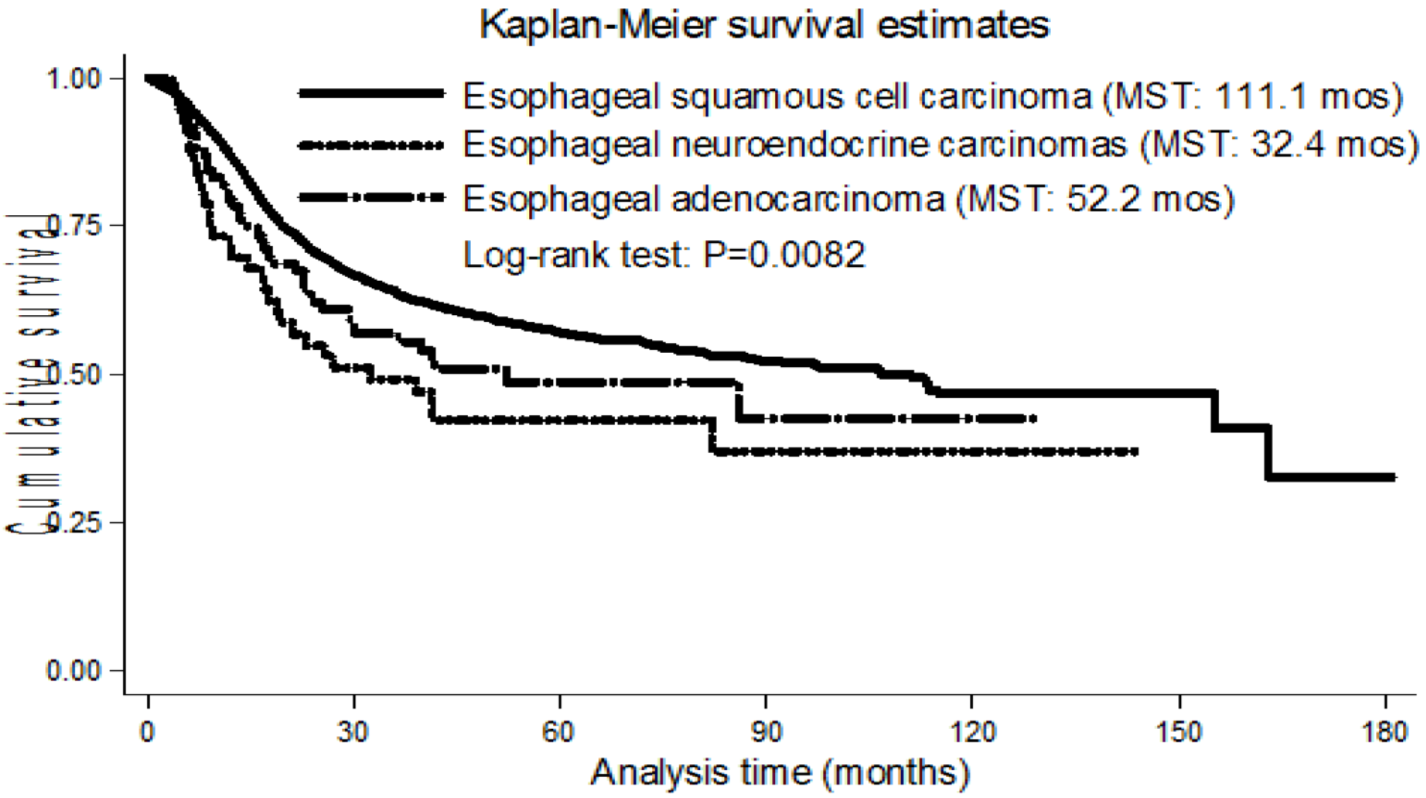

Abbreviations: MST, median survival time; mos, months.

Supplementary Figure S1. Kaplan-Meier survival estimates by histological types of esophageal cancer. Survival difference of three histological types was identified by the Log-rank test.
